# Ibrutinib plus Obinutuzumab as Frontline Therapy for Chronic Lymphocytic Leukemia Is Associated with a Lower Rate of Infusion-Related Reactions and with Sustained Remissions after Ibrutinib Discontinuation: A Single-Arm, Open-Label, Phase 1b/2 Clinical Trial NCT0231576

**DOI:** 10.1155/2022/4450824

**Published:** 2022-01-22

**Authors:** Januario E. Castro, Paula A. Lengerke-Diaz, Juliana Velez Lujan, Michael Y. Choi, Eider F. Moreno-Cortes, Jose V. Forero, Juan Esteban Garcia-Robledo, Chaja Jacobs, Colin McCarthy, Alaina Heinen, Carlos I. Amaya-Chanaga, Thomas J. Kipps

**Affiliations:** ^1^Division of Hematology and Medical Oncology, Mayo Clinic, Phoenix, AZ, USA; ^2^CLL Research Consortium (CRC), UCSD, San Diego, CA, USA; ^3^Moores Cancer Center, UCSD, La Jolla, CA, USA; ^4^Eisai Inc., Woodcliff Lake, Bergen, NJ, USA

## Abstract

Ibrutinib-based therapies are costly and require continuous administration. We hypothesized combining BTK inhibition with anti-CD20 monoclonal antibodies would yield deep remissions allowing discontinuation. We enrolled 32 therapy-naïve CLL patients to receive ibrutinib plus obinutuzumab, followed by single-agent ibrutinib. Patients could discontinue ibrutinib after 36 months with sustained complete response (CR). We evaluated treatment safety, efficacy, and outcomes after ibrutinib discontinuation. The overall response rate was 100%, 28% achieved a CR, and 12.5% achieved bone marrow undetectable minimal residual disease. At a three-year median follow-up, 91% remain in remission with 100% overall survival. Five patients in sustained CR stopped ibrutinib and have not progressed. Eight non-CR patients discontinued for other reasons, with only two progressing. The treatment was safe, with a lower IRR rate. All patients responded to treatment with longer time-to-progression after discontinuation of ibrutinib. Our data support the evaluation of ibrutinib discontinuation strategies in more extensive clinical trials (https://Clinicaltrials.gov Identifier https://clinicaltrials.gov/ct2/show/NCT02315768).

## 1. Introduction

Standard treatment for patients with chronic lymphocytic leukemia (CLL) includes the combination of monoclonal antibodies (mAbs) and targeted therapies with small molecule inhibitors. The phase 3 trial RESONATE-2 showed that single-agent ibrutinib, a first-in-class Bruton tyrosine kinase (BTK) inhibitor, is effective in previously untreated patients, including those that are older than 65 or considered unfit to receive chemotherapy. This was supported by a significant benefit in progression-free survival (PFS), and overall survival (OS) compared to chlorambucil [1]. The majority of patients treated with single-agent ibrutinib achieve an objective response with prolonged PFS that exceeds five years of duration [2, 3]. However, the number of patients with complete response (CR) is low (4% at a median follow-up of 18.4 months); patients commonly experience increasing lymphocytosis that can be confused with disease progression and require continuous administration of this medication to prevent leukemia progression [1].

Randomized trials show that the addition of type II glycoengineered anti-CD20 monoclonal antibodies (e.g., obinutuzumab) can increase the efficacy, CR rates, and the duration of response when combined with different agents (chlorambucil, ibrutinib, and venetoclax) [4–6]. Moreover, BTK inhibition with ibrutinib induces the redistribution of tissue-resident CLL cells into the peripheral blood and makes them an ideal target for anti-CD20 mAbs like obinutuzumab [7–9].

We hypothesized that combining BTK inhibition with anti-CD20 mAbs would yield deep remissions allowing discontinuation of ibrutinib. We conducted a phase 1b/2 clinical study to evaluate whether the combination of ibrutinib and obinutuzumab-Gazyva® (IG-regimen) is safe and effective in previously untreated CLL patients older than 65 or considered unfit/unwilling to receive chemoimmunotherapy. In addition, our study was designed to evaluate the option to discontinue ibrutinib after three years of treatment in patients with sustained CR.

## 2. Methods

### 2.1. Study Design and Participants

This phase 1b/2, single-arm, open-label trial enrolled therapy-naïve adult patients with CLL requiring treatment by International Workshop on Chronic Lymphocytic Leukemia (iwCLL) 2008 criteria. Eligible patients were ≥65 years, or if younger, they must refuse or be considered unsuitable for standard chemoimmunotherapy due to the presence of comorbidities.

Patients had a performance status of 2 or less, and normal renal and hepatic function. We excluded patients with active or symptomatic infection, including hepatitis B-C or human immunodeficiency virus, as well as pregnant or nursing women. All the patients provided written informed consent for this protocol, which was approved by the University of California San Diego (UCSD) Human Research Protections Program and conducted following the Declaration of Helsinki. The first six patients were included in the phase 1b analysis. Since no dose-limiting toxicity was observed during the first cycle, we proceeded with enrollment of 26 additional patients for phase 2 of the study.

### 2.2. Procedures

All patients received ibrutinib plus obinutuzumab. The IG-regiment consisted of oral ibrutinib (420 mg once daily—started one hour before antibody infusion) administered until disease progression, unacceptable toxicity, or other reason for treatment discontinuation. At the 3-year follow-up, patients that maintained a complete response based on clinical and hematological criteria underwent additional CT scans (chest, abdomen, and pelvis) and bone marrow biopsy, and if they had a confirmed CR status, they were given the option to stop ibrutinib or switch to the commercial supply of this medication. All other patients without signs of disease progression were recommended to continue ibrutinib indefinitely and were switched to commercial supply.

Obinutuzumab was administered intravenously as follows: 100 mg on day 1, 900 mg on day 2, 1,000 mg on day 8 and day 15 of cycle 1, and 1,000 mg on day 1 of subsequent 28-day cycles, for a total of six cycles. All patients received prophylaxis for tumor lysis syndrome and premedication to reduce the risk of infusion-related reactions (IRR), which included hydration, allopurinol, intravenous glucocorticoid (dexamethasone or methylprednisolone), oral acetaminophen, and antihistamine. Patients were withdrawn from the study when they received a new treatment due to disease progression, upon withdrawal of their consent, loss of follow-up, or death.

We collected serial peripheral blood samples before, during, and after obinutuzumab infusion to perform cytokine/chemokine analyses using Luminex technology. The detailed methods were described previously [10]. This analysis focused on the cytokine/chemokine kinetics before, during, and after the first cycle on day 1 of obinutuzumab infusion.

Two months after completing the last obinutuzumab infusion, the patients underwent a response assessment following the 2008 iwCLL guidelines. Afterward, the patients were followed every three months for nine months and every six months until the end of the study. CT scans were performed and assessed by a local radiologist at baseline and during the response assessment period.

### 2.3. Statistical Analysis

We used the overall response rate (ORR) as the primary endpoint for sample size calculation based on the response previously reported with chlorambucil combined with obinutuzumab [4]. We hypothesized that ibrutinib combined with obinutuzumab would induce an ORR of 94%, which is 16% higher than the combination with chlorambucil [4].

We used Simon's two-stage optimal design [11]. The null hypothesis that the true ORR is 78% was tested against a one-sided alternative; 29 or more responses in 32 patients yielded to 70% power to detect an ORR of 94% with a type I error rate of 5%. PFS, treatment-free survival (TFS), and overall survival (OS) were analyzed using the Kaplan–Meier method. Time intervals were measured from the first day of treatment until disease progression, time-to-next-treatment per iwCLL guidelines, or death. Patients were followed up until initiation of a new treatment, consent withdrawal, loss of follow-up, or death. For subjects that have maintained a CR (based on clinical and hematological assessments), an additional bone marrow biopsy and aspirate were performed after three years of completion of study treatment in order to assess MRD and discuss the possibility of ibrutinib discontinuation.

Patient demographics, baseline characteristics, adverse events, and efficacy outcomes were summarized using frequencies and corresponding percentages. According to data distribution, continuous variables were summarized using either mean with standard deviation (SD) or median with interquartile range (IQR). A 2-sided *p* value of less than 0.05 was considered significant. Analyses were performed using GraphPad Prism (version 8.2.1).

### 2.4. Role of the Funding Source

The study was supported by funding and drug supply (ibrutinib) from Pharmacyclics, an AbbVie Company. The pharmaceutical sponsor approved the study design and reviewed the manuscript but had no role in the collection or analyses of data or the manuscript's writing. All authors had full access to all the data in the study and had final responsibility for submitting for publication.

## 3. Results

### 3.1. Patient Characteristics

**Figure 1:**
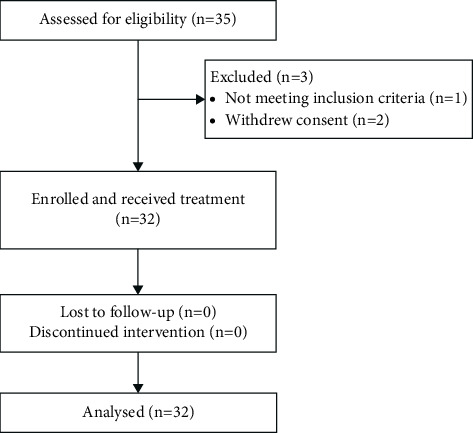
Study enrollment and patient flow. Diagram of the 35 patients screened for eligibility. One patient was ineligible due to Richter's transformation during screening period. Two patient's voluntary withdrew consent prior to intervention.

Thirty-five patients were screened, and 32 eligible patients were enrolled to receive the IG-regimen (Figure 1). Patient baseline characteristics are listed in Table 1. The median age was 66 years (IQR 59–73), and 6% of the evaluated patients (*n* = 2) had 17p deletion. The median follow-up was 35.5 months (IQR 24.5–42.7).

### 3.2. Safety and Tolerability

All patients completed the six planned cycles of obinutuzumab. At a median follow-up of 35.5 months after starting treatment, thirteen patients (41%) discontinued ibrutinib. The median time to ibrutinib discontinuation was 35 months (95% CI 26–41).

During the initial 18 months of follow-up, only three patients (9%) discontinued ibrutinib due to adverse events, one of them due to grade 2 atrial fibrillation, and the remaining two because of grade 1-2 skin rash. No treatment-related adverse events leading to obinutuzumab discontinuation were observed.

The most common adverse events (>5% CTCAE grade 3-4) were neutropenia, thrombocytopenia, and hyperglycemia. IRR of any grade occurred in 19% of the patients, and grade 3 or 4 IRR occurred in 3% of patients (Table 2). None of the patients required hospitalization or discontinued obinutuzumab because of IRR.

We performed a detailed correlative analysis of cytokines/chemokines associated with IRR, and we recently published the results [10]. The data shows that, in most patients, the administration of ibrutinib before the infusion of obinutuzumab abrogates the release of cytokines/chemokines expected with this antibody, and this lack of immune mediators correlated directly with the absence or reduction in IRR [12]. Moreover, the few patients who developed IRR had a cytokine/chemokine profile similar to the one observed after administering single-agent obinutuzumab [12]. After the obinutuzumab infusion, we found that CCL3, IFN-*γ*, and TNF-*α* showed levels with a significant increase in patients that developed IRR. CCL4 reached the highest biomarker peak in patients with IRR compared to patients without it; however, this difference was not statistically significant.

Also, our data suggest that high preinfusion levels of CCL3, IFN-*γ*, and IL-6 may have a predictive role in identifying patients that are likely to develop IRR [10]. Overall, these analyses suggest that ibrutinib administered 1 hour before the first dose of obinutuzumab (100 mg in day 1, cycle 1) can abrogate IRR's clinical manifestations and reduce the cytokine/chemokine release associated with immune effector activation triggered by this antibody.

### 3.3. Efficacy

All patients treated achieved an objective response (ORR 100%) based on the iwCLL 2008 criteria (Table 3). We performed an updated analysis applying iwCLL 2018 criteria, with consistent results.

**Figure 2:**
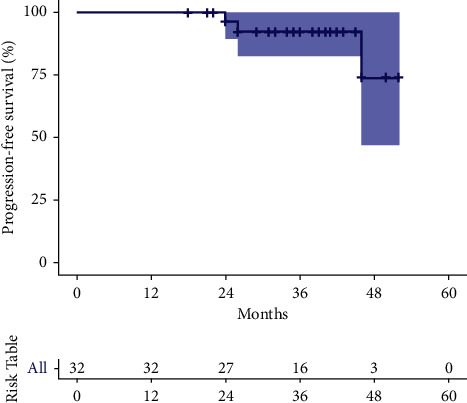
Kaplan–Meier analysis of progression-free survival (PFS).

**Figure 3:**
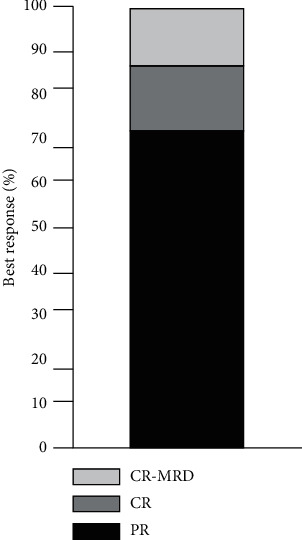
Best response achieved.

The overall response rate was 100%; nine patients (28%) achieved a CR, and four patients (12.5%) achieved undetectable minimal residual disease (uMRD) in the bone marrow, defined as <10-4 CLL cells on multicolor flow cytometry (Figure 2). At a median follow-up of 35.5 months (95% CI 26–41) after starting treatment, 91% of the enrolled patients remain in remission, the median PFS has not been reached, and the cohort has a 100% overall survival (Table 4, Figure 3).

The median baseline absolute lymphocyte count (ALC) was 59 × 109/L (IQR 21-105). All patients showed a rapid decline in lymphocytosis, and none of them showed ibrutinib-induced lymphocytosis. Twenty-six (88%) patients achieved an ALC <4 × 109/L during the first cycle of treatment, and four patients (12%) required more than three cycles to achieve an ALC complete response.

**Figure 4:**
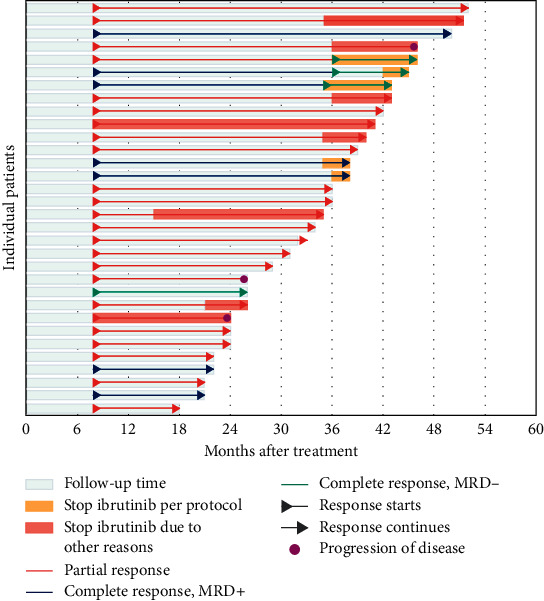
Swimmers plot of patients enrolled. This figure provides a snapshot of all patients enrolled in the study that received medication. Each bar represents one subject in the study. Patients started treatment at time point zero. First response assessment occurred eight months after initiations of therapy according yo iWCLL 2018 guidelines.

Sixteen patients (50%) have completed a long-term follow-up of 36 months. Six patients showed CR, with three of them achieving uMRD in the bone marrow. Ten of these patients were in PR at 36 months, and only one of them had progressed and started treatment for symptomatic stage I disease with obinutuzumab plus venetoclax (Figure 4).

In total, thirteen patients (41%) have stopped ibrutinib, with a median discontinuation time of 35 months. Five (16%) of these patients had CRs and discontinued ibrutinib after 36 months per-protocol guideline. Eight additional patients (25%) had PRs. They discontinued ibrutinib without being eligible based on the protocol: four patients discontinued before 36 months due to toxicities, and four patients discontinued after 36 months (2 due to side effects, and 2 due to a financially driven decision). One patient eligible to discontinue ibrutinib decided to remain on treatment despite sustained CR. After a median follow-up time following ibrutinib discontinuation of 8 months (IQR 3.5–17), only two out of 13 patients have progressed (10 and 17 months after ibrutinib discontinuation). None of the five patients that stopped ibrutinib after achieving a CR have shown signs of disease progression (Figure 4).

## 4. Discussion

This study shows that obinutuzumab, in combination with ibrutinib as a frontline treatment for older or unfit CLL patients, is safe, well-tolerated, and effective. Moreover, it successfully explores the possibility of fixed-duration ibrutinib in a subset of patients with deep and sustained responses.

Our safety data is encouraging, with lower rates of infusion-related reactions observed with the IG-regimen (obinutuzumab plus ibrutinib) in comparison to historical data from CLL11 study (obinutuzumab plus chlorambucil). Our overall IRR rate was 19% vs. 65%, and only 3% of patients had grade 3-4 IRR vs. 20% in the CLL11. None of our patients required hospitalization or discontinuation of obinutuzumab therapy due to IRR or any other causes, while 10% of the patients withdrew from the CLL11 study due to IRR [4]. Additionally, the iLLUMINATE study (obinutuzumab plus ibrutinib) found a similar safety profile to ours (overall IRR rate of 25%, and 3% grade 3-4 IRR) [6].

Our correlative analyses show how ibrutinib abrogates the release of cytokines such as IFN-*γ* and TNF-*α*, which have shown to be responsible for the clinical manifestations associated with IRR [12, 13]. Importantly, we found that patients with IRR showed a higher peak of CCL3 and CCL4. These chemokines are associated with tumor burden, poor prognosis, and release of other inflammatory factors that contribute to the survival of CLL cells [14, 15]. Moreover, the downregulation effect on cytokine/chemokine release and IRR did not seem to impact the response of patients to either ibrutinib or obinutuzumab. Our data suggest that the underlying mechanism responsible for the blockade of immune modulators is most likely ibrutinib and its BTK mediated inhibition of critical pathways responsible for immune activation.

The overall safety profile of the IG-regimen was consistent with the known profiles of the individual drugs, with no new adverse events identified. The most frequent grade 3-4 adverse events were neutropenia, thrombocytopenia, and hyperglycemia. Our high rate of hyperglycemia is likely due to the intravenous administration of glucocorticoids as premedication.

Regarding efficacy, our investigator assessment showed an overall response rate of 100%, and 9 out of 32 patients (28%) had a CR. These results are comparable with the independent review committee-based assessment in the iLLUMINATE study (88% and 19%, respectively). However, we recognize the potential limitations of our study, including the sample size and single-arm design. We observed a rate of uMRD in the bone marrow of 12.5%, in comparison with the 20% observed in the iLLUMINATE study and the 19.5% in the CLL11 trial. However, in the CLL11 study, the uMRD rate used a denominator of the number of patients from whom we got the result rather than the total number of patients treated. Also, in the iLLUMINATE trial some uMRD occurred in patients with partial (including nPR) responses, while we only systematically assessed MRD in patients with CR.

Our study included an innovative design, in which patients maintaining a CR response at 3-year follow-up were recommended to stop ibrutinib. The rationale for this design is that patients with sustained CR could have similar outcomes with interruption of ibrutinib therapy, decreasing the risk of adverse events, serious complications, toxicities, in addition to reducing the financial burden that is associated with long-term ibrutinib management. This hypothesis is supported by observations, in which patients that discontinued ibrutinib due to side effects had better outcomes than those that stopped therapy because of disease progression [16, 17].

At a median follow-up of 35.5 months, overall ibrutinib discontinuation in our study was at 41%. This discontinuation rate is higher than the iLLUMINATE study (30%, median follow-up of 31 months) [6] but is lower than pooled clinical trial analyses (51%, median follow-up of 40 months) [18] and real-world data (41%, median follow-up 17 months) [19]. Of notice, in our study, only three patients (9%) discontinued ibrutinib during the first 18 months.

The response status at the time of discontinuation appears to determine the long-term outcome of patients that discontinue ibrutinib. In our trial, five patients with sustained CR decided to discontinue ibrutinib, and at the time of this report, they have been on clinical follow-up for up to 10 months without signs of disease progression [Median follow-up 3.3 months (95% CI 2–10)]. Additionally, 8 patients with PR have discontinued ibrutinib, and only 2 have disease progression (median follow-up 14 months (95% CI 5–34)). Overall, in our cohort, patients that stop ibrutinib for any reason (*n* = 13) have a longer time-to-progression, which exceeds historical data (13.5 months vs. 2.7 months) with a median follow-up time of 8 months after ibrutinib discontinuation, and a median time to progression that has not been reached [16].

We acknowledge that our study has limitations that prevent a direct comparison with historical data on ibrutinib discontinuation, including the small number of patients in CR who stopped ibrutinib and the relatively short follow-up from stopping ibrutinib. However, our data after discontinuation of ibrutinib is highly encouraging in comparison to the real-world studies that report 2.7 months as the median time to next therapy among patients who stopped ibrutinib (6.5 months for those who stopped ibrutinib due to toxicity versus 0.3 months for those who stopped due to disease progression—CLL progression or Richter's transformation) [16]. Additional data regarding progression after ibrutinib discontinuation in patients with sustained remission is limited, and studies such as the CAPTIVATE trial (NCT# https://clinicaltrials.gov/ct2/show/NCT03462719) will help confirm whether ibrutinib, in combination with other agents, could induce deep responses that allow patients to discontinue long-term treatment without compromising disease-free survival.

Overall, we show that the combination of ibrutinib and obinutuzumab as first-line treatment in CLL patients that either refuse or were considered unsuitable for standard chemoimmunotherapy is safe, is highly effective, and induces deep responses associated with uMRD. The IG-regimen was associated with a lower rate of infusion-related reactions, abrogation in the release of associated cytokines/chemokines, and sustained remissions despite ibrutinib discontinuation. Our observations are encouraging and warrant confirmation in long-term prospective studies.

## Figures and Tables

**Table 1:** Descriptive characteristics of the study population, means ± SD, median [IQR], or *N* (%).

Characteristic	Ibrutinib plus obinutuzumab
*N*	32
Age (years)	66 [59–73]
65 years or older (%)	17 (53)
Male	18 (56)
*Cumulative illness rating scale*	
Total score	9 [6–11]
Greater than 6 (%)	23 (72)
*ECOG performance status*	
0	16 (50)
1	10 (31)
2	6 (19)
*Calculated creatinine clearance*	
Below 60 mL/min	9 (28)
*Rai stage*	
0–II	18 (56)
III-IV	14 (44)
*Lymph nodes*	
Median SPD, cm^2^ (min-max)	17 (7–361)
Bulky disease ≥5 cm	6 (19)
*Cytogenetics*	
Del13q	19 (59)
Trisomy 12	9 (28)
Del11q	6 (19)
Del17p	2 (6)
Unmutated IgVH	11/18 (61)
High ZAP-70 expression	10/28 (36)
*Hematological parameters*	
Hemoglobin, g/dL	12 ± 2
Platelets × 10^9^/L	147 ± 66
Neutrophils × 10^9^/L	4 [3–5]
Lymphocytes × 10^9^/L	59 [21–105]

**Table 2:** Adverse events.

	Number of patients (%) Ibrutinib plus obinutuzumab (*n* = 32)	
	Any grade	Grade 3-4
All	32 (100)	25 (78)

*Nonhematological*		
Hyperglycemia	23 (72)	2 (6)
AST increased	16 (50)	1 (3)
Diarrhea	14 (44)	1 (3)
Infections (skin, lung, and eye)	7 (22)	1 (3)
Hyponatremia	7 (22)	1 (3)
Hypocalcemia	7 (22)	1 (3)
Blood bilirubin increased	11 (34)	0 (0)
Fever	11 (34)	0 (0)
Fatigue	11 (34)	0 (0)
Hyperphosphatemia	10 (31)	0 (0)
ALT increased	10 (31)	0 (0)
Hyperkalemia	8 (25)	0 (0)
Creatinine increased	6 (19)	0 (0)
Headache	6 (19)	0 (0)
Flu-like symptoms	6 (19)	0 (0)
Arthralgia	5 (16)	0 (0)
Alkaline phosphatase increased	4 (12)	0 (0)
Back pain	4 (12)	0 (0)
Neck pain	3 (9)	0 (0)
Atrial fibrillation	2 (6)	0 (0)
*Hematological*		
Neutropenia	18 (56)	11 (34)
Thrombocytopenia	25 (75)	8 (25)
Anemia	8 (25)	0 (0)

**Table 3:** Summary of the results.

	Number of patients (%) Ibrutinib plus obinutuzumab (*n* = 32)
*Primary endpoints*	
ORR	32 (100)
*Secondary endpoints*	
PFS	Not reached
TFS	Not reached
OS	32 (100)
CR	9 (28.5)
MRDneg	4 (12.5)
PR	20 (62)
SD	0 (0)
PD	3 (9)

**Table 4:** Infusion related reactions.

	Number of patients (%) Ibrutinib plus obinutuzumab (*n* = 32)
Infusion related reaction, all grades	6 (19)
Infusion related reaction, grade 3 or 4	1 (3)
Treatment discontinuation due to an infusion related reaction	0 (0)

## Data Availability

The data used to support the findings of this study are available from the corresponding author upon request.

## References

[1] Burger J. A., Tedeschi A., Barr P. M. (2015). Ibrutinib as initial therapy for patients with chronic lymphocytic leukemia. *New England Journal of Medicine*.

[2] Woyach J. A., Ruppert A. S., Heerema N. A. (2018). Ibrutinib regimens versus chemoimmunotherapy in older patients with untreated CLL. *New England Journal of Medicine*.

[3] Burger J. A., Barr P. M., Robak T. (2019). Long-term efficacy and safety of first-line ibrutinib treatment for patients with CLL/SLL: 5 years of follow-up from the phase 3 RESONATE-2 study. *Leukemia*.

[4] Goede V., Fischer K., Busch R. (2014). Obinutuzumab plus chlorambucil in patients with CLL and coexisting conditions. *New England Journal of Medicine*.

[5] Fischer K., Al-Sawaf O., Bahlo J. (2019). Venetoclax and obinutuzumab in patients with CLL and coexisting conditions. *New England Journal of Medicine*.

[6] Moreno C., Greil R., Demirkan F. (2019). Ibrutinib plus obinutuzumab versus chlorambucil plus obinutuzumab in first-line treatment of chronic lymphocytic leukaemia (iLLUMINATE): a multicentre, randomised, open-label, phase 3 trial. *The Lancet Oncology*.

[7] Pavlasova G., Borsky M., Seda V. (2016). Ibrutinib inhibits CD20 upregulation on CLL B cells mediated by the CXCR4/SDF-1 axis. *Blood*.

[8] Skarzynski M., Niemann C. U., Lee Y. S. (2016). Interactions between ibrutinib and anti-CD20 antibodies: competing effects on the outcome of combination therapy. *Clinical Cancer Research*.

[9] Wodarz D., Garg N., Komarova N. L. (2014). Kinetics of CLL cells in tissues and blood during therapy with the BTK inhibitor ibrutinib. *Blood*.

[10] Velez Lujan J., Lengerke-Diaz P. A., Jacobs C. (2019). Ibrutinib reduces obinutuzumab infusion related reactions in patients with chronic lymphocytic leukemia and is associated with changes in plasma cytokine levels. *Haematologica*.

[11] Simon R. (1989). Optimal two-stage designs for phase II clinical trials. *Controlled Clinical Trials*.

[12] Freeman C. L., Morschhauser F., Sehn L. (2015). Cytokine release in patients with CLL treated with obinutuzumab and possible relationship with infusion-related reactions. *Blood*.

[13] Niemann C. U., Biancotto A., Chang B. Y. (2013). Cytokine and T-cell phenotypic changes upon in vivo ibrutinib therapy for CLL—targeting both CLL cells and the tumor-microenvironment. *Blood*.

[14] Sivina M., Hartmann E., Krupnik D. (2009). CCL3 and CCL4 plasma levels correlate with established prognostic markers in chronic lymphocytic leukemia: towards a simple, ELISA-based assay for risk assessment. *Blood*.

[15] Tolbah F., Kamal H., El-behisy M., El-Sakhawy Y., Abd-El Latif S. (2016). The prognostic role of chemokine ligand-3 in chronic lymphocytic leukemia. *The Egyptian Journal of Haematology*.

[16] Hampel P. J., Chaffee K. G., Ding W. (2018). Rapid progression of disease following ibrutinib discontinuation in patients with chronic lymphocytic leukemia. *Journal of Clinical Oncology*.

[17] Jain P., Keating M., Wierda W. (2015). Outcomes of patients with chronic lymphocytic leukemia after discontinuing ibrutinib. *Blood*.

[18] Woyach J. A., Ruppert A. S., Guinn D. (2017). BTKC481S-mediated resistance to ibrutinib in chronic lymphocytic leukemia. *Journal of Clinical Oncology*.

[19] Mato A. R., Nabhan C., Thompson M. C. (2018). Toxicities and outcomes of 616 ibrutinib-treated patients in the United States: a real-world analysis. *Haematologica*.

